# Tepotinib for advanced non-small-cell lung cancer with *MET* exon 14 skipping mutations

**DOI:** 10.1016/j.esmoop.2022.100449

**Published:** 2022-03-22

**Authors:** M. Błaszkowska, Z. Specht-Szwoch, R. Dziadziuszko

**Affiliations:** 1 Early Clinical Trials Centre, Medical University of Gdańsk, Gdańsk, Poland; 2 Department of Oncology and Radiotherapy, Medical University of Gdańsk, Gdańsk, Poland

On 16 December 2021, the Committee for Medicinal Products for Human Use (CHMP) issued a positive opinion, recommending a marketing authorisation for tepotinib (Tepmetko, Merck KGaA, Darmstadt, Germany), an oral, highly selective mesenchymal–epithelial transition (MET) inhibitor.^1^ This recommendation is intended for non-small-cell lung cancer (NSCLC) patients, whose tumours harbour *MET* gene exon 14 (METex14) skipping alterations. The CHMP statement follows Food and Drug Administration-accelerated approval of tepotinib for the same indication, issued on 3 February 2021.^2^

*MET* gene encodes hepatocyte growth factor receptor (HGF), involved in the regulation of cell proliferation, migration, invasion and angiogenesis. Activation of MET tyrosine kinase activity leads to epithelial to mesenchymal cell transition, a process associated with cancer invasiveness, increased metastatic potential and resistance to therapy. *MET* gene is deregulated in a proportion of NSCLCs via three distinct mechanisms: exon 14 skipping mutations (resulting in decreased MET degradation in the process of ubiquitination and increased protein stability in the cell membrane; ∼3% of patients); *MET* amplification (∼2%-5% of patients *de novo*, relatively common as a mechanism of acquired resistance to targeted therapies such as epidermal growth factor receptor, anaplastic lymphoma kinase, ROS proto-oncogene 1 or rearranged during transfection inhibitors); and *MET* gene fusions (rarely reported). METex14 aberrations occur typically in older patients, relatively frequently smokers and are associated with sarcomatoid histology.^3^

Tepotinib is an oral, highly selective kinase inhibitor that targets MET kinase activity, including the isoform produced by exon 14 skipping mutations. The recommended dose is 450 mg once daily with food (two 225 mg tablets). Tepotinib inhibits both HGF-dependent and -independent MET kinase activity, which results in greater suppression of MET signalling as compared to inhibitors of only ligand-dependent activity.^4^ The pharmacokinetic interaction of tepotinib with ATP-binding cassette transporters and cytochrome *P*-450 (CYP) drug-metabolising enzymes as well as their possibility to combat multidrug resistance were explored *in vivo* and *in vitro*.^5^ Metabolism and *in vitro* data showed that tepotinib is a substrate of CYP3A4, CYP2C8 and P-glycoprotein (P-gp). However, it is not possible to accurately quantify the fractions metabolised by the individual major CYP isoenzymes.^6^

Toxicology studies show that the main target organs in animal studies based on tepotinib-related histopathological changes included liver (significant bile duct hyperplasia), lung and gastrointestinal tract, with occasional evidence of oedema in some organs.^6^

It is recommended that patient taking tepotinib should avoid using concomitant medications with strong CYP3A and CYP3A4 inducers and with dual strong inhibitors of CYP 3 or P-gp.^6^

Brain penetration and observed efficacy of tepotinib in NSCLC METex14 patients with brain metastases have been confirmed in rat studies. The calculated ratio of unbound brain to plasma was 0.25, indicating a brain penetration which is sufficient for inhibition of the intracranial disease.^7^

Tepotinib recommendation, solely for patients with tumours showing METex14 skipping mutations, is based on the results of Vision (NCT02864992), a single-arm, open-label global study.^8^ *MET* gene abnormalities were identified prospectively by tissue- or plasma-based PCR or next-generation sequencing assays. Patients (*n* = 152, 69 treatment naive and 83 previously treated) enrolled in the Vision trial were relatively older (median age of 73 years), mostly of white ethnicity, with equal distribution of sex, and never-smoking history (less than 100 cigarettes in a lifetime) was noted in 45% of subjects. Independent review committee objective response rate, assessed by RECIST 1.1 in patients with at least 9 months of follow-up, was 46% [primary endpoint, 95% confidence interval (CI): 36% to 57%], with a median duration of response of 11.1 months (95% CI: 7.2 months-not evaluable).

In the safety population of 152 patients with tumours showing METex14 skipping alterations or *MET* amplification, most common grade 3 or higher adverse events were reported in 28% of patients, with peripheral oedema and pleural effusion being the most common, followed by lipase, amylase or liver transaminase increase. Serious adverse events related to tepotinib were noted in 15% of patients, and included mostly events associated with oedema. Dose reduction was undertaken in 15% and permanent treatment discontinuation in 11% of patients, mostly due to peripheral oedema, pleural effusion or dyspnoea. The most common grade 1 or 2 adverse events include peripheral oedema, nausea/vomiting, diarrhoea, creatinine, lipase, amylase or transaminase increase and asthenia/fatigue.

Positive CHMP recommendation of tepotinib for treatment of NSCLC patients with METex14 skipping alterations adds to the enlarging portfolio of targeted therapies in lung cancer and is in line with the National Comprehensive Cancer Network recommendations for the diagnosis and treatment of advanced NSCLC.^9^ Depending on the European Medicines Agency’s approval and reimbursement decisions on the country level, patients in Europe will now have access to novel important targeted agent if occurrence of METex14 skipping mutation is detected. Positive efficacy data of tepotinib and another highly selective MET inhibitor, capmatinib,^6^ prompt several further steps: firstly, molecular testing for exon 14 skipping alterations should now become an obligatory standard diagnostic procedure, optimally with next-generation multigene panel testing upon lung cancer diagnosis; secondly, efficacy of tepotinib and capmatinib should be investigated in early NSCLC, potentially in the adjuvant setting; thirdly, efficacy in other important MET aberrations, such as *MET* amplification (*de novo* or as a resistance mechanism to other targeted therapies), or rare *MET* gene rearrangements, should further be explored, in NSCLC and also in other tumour types; fourthly, mechanisms of resistance to tepotinib and capmatinib should be determined to rationally design subsequent therapies.
